# Isolation and characterization of Arctic microorganisms decomposing bioplastics

**DOI:** 10.1186/s13568-017-0448-4

**Published:** 2017-07-11

**Authors:** Aneta K. Urbanek, Waldemar Rymowicz, Mateusz C. Strzelecki, Waldemar Kociuba, Łukasz Franczak, Aleksandra M. Mirończuk

**Affiliations:** 1 Department of Biotechnology and Food Microbiology, Wroclaw University of Environmental and Life Sciences, Chełmońskiego 37, 51-630 Wrocław, Poland; 2 Institute of Geography and Regional Development, University of Wroclaw, pl. Uniwersytecki 1, 50-137 Wrocław, Poland; 3 Faculty of Earth Sciences and Spatial Management, Maria Curie-Sklodowska University, al. Krasnicka 2 CD, 20-718 Lublin, Poland

**Keywords:** Biodegradation, Biodegradable plastics (BP), Arctic microorganisms, Microbial degradation

## Abstract

The increasing amount of plastic waste causes significant environmental pollution. In this study, screening of Arctic microorganisms which are able to degrade bioplastics was performed. In total, 313 microorganisms were isolated from 52 soil samples from the Arctic region (Spitsbergen). Among the isolated microorganisms, 121 (38.66%) showed biodegradation activity. The ability of clear zone formation on emulsified poly(butylene succinate-co-adipate) (PBSA) was observed for 116 microorganisms (95.87%), on poly(butylene succinate) (PBS) for 73 microorganisms (60.33%), and on poly(ɛ-caprolactone) (PCL) for 102 microorganisms (84.3%). Moreover, the growth of microorganisms on poly(lactic acid) (PLA) agar plates was observed for 56 microorganisms (46.28%). Based on the 16S rRNA sequence, 10 bacterial strains which showed the highest ability for biodegradation were identified as species belonging to *Pseudomonas* sp. and *Rhodococcus* sp. The isolated fungal strains were tested for polycaprolactone films and commercial corn and potato starch bags degradation under laboratory conditions. Strains 16G (based on the analysis of a partial 18S rRNA sequence, identified as *Clonostachys rosea*) and 16H (identified as *Trichoderma* sp.) showed the highest capability for biodegradation. A particularly high capability for biodegradation was observed for the strain *Clonostachys rosea*, which showed 100% degradation of starch films and 52.91% degradation of PCL films in a 30-day shake flask experiment. The main advantage of the microorganisms isolated from Arctic environment is the ability to grow at low temperature and efficient biodegradation under this condition. The data suggest that *C. rosea* can be used in natural and laboratory conditions for degradations of bioplastics.

## Introduction

Three hundred and eleven tons of plastic were produced in 2014 (PlasticsEurope 2015). This enormous number suggest that plastic has many applications in our daily life and industries (Uchida et al. 2000), mainly in packaging, building and construction, automotive, agriculture, electrical and electronic (PlasticsEurope 2015). However, plastic materials have some disadvantages, the most important criterion is its long long-term persistence in the environment in consequence of their resistance to degradation (Gajendiran et al. 2016). Extensive use of polymeric materials have made plastic pollution as significant environmental issue (Guo et al. 2012) and represents a major threat to ecological systems (Shah et al. 2014). Due to accumulation of plastics in the environment, mainly in open waters, 1 million seabirds and 100 thousand marine mammals die annually. 44% of all seabirds, 86% of all sea turtles and 43% of all marine mammal species and many fish have been affected by entanglement or ingestion of marine debris (Allsopp et al. 2006).

The Arctic region is also affected by the problem of the pollution. Despite the fact that Spitsbergen is located far away from the closet settlements, the coast is not free from human’s influence. Among the large amounts of foreign material, mostly wood, glass and plastic bottles, tins, bulbs, pieces of clothes, bags, pieces of plastic tape, fishing nets and ropes were found (Czubla 1994). The continuously growing global production of plastic has a big influence for this virgin territory. The plastic ingestion has been documented in over 100 species of seabird (Trevail et al. 2015).

Therefore researchers have been searching for new, alternative materials which can be used as good substitutes for conventional plastics (Emadian et al. 2016). One of them is production of biobased and biodegradable plastics [BP]. Nowadays, bioplastics constitute about one per cent of the about 300 million tons of plastic produced annually. Production of bioplastics in 2014 reached approximately 2 million tons globally; moreover, it is predicted that in 2019 production will reach almost 8 million tons (Bioplastics 2015). Generally, BP can be divided in two groups, renewable resource-based polymers and petroleum-based polymers (Penkhrue et al. 2015). The first group is derived from renewable resources (Guo et al. 2012) such a biomass of organic waste material or crops (Kershaw 2015), it can be produced by microorganisms or obtained from genetically modified plants (Shah et al. 2008). Polyhydroxyalkanoates (PHAs) and poly(lactic acid) (PLA) are renewable source-based polymers. Polycaprolactone (PCL), and poly(butylenes succinate) (PBS) are petroleum based. Despite this fact, they can be degraded by microorganisms s in the natural environment (Koitabashi et al. 2012; Li et al. 2012; Penkhrue et al. 2015). In this process, known as biodegradation, compounds commonly occurring in the natural environment (Bhardwaj et al. 2012), such as CO_2_, H_2_O, NH_4_, N_2_, H_2_ and biomass are produced. Therefore bioplastics do not negatively affect the natural environment. Biodegradation is the partial or complete hydrolysis of a polymer by microbial activity with the positive influence of photodegradation (Kershaw 2015). However, this process can also be time consuming and often high temperature is required. For example, it has be confirmed that high temperature (60 °C) is the best for PLA degradation (Prema et al. 2013; Sukkhum et al. 2009). Moreover 20 months were required to decomposition of PLA in soil (Urayama et al. 2002) in contrast to the compost, where degradation was lasted 45–60 days. Other biodegradable plastics are more susceptible to microbial attack in the natural environment than PLA (Tokiwa and Calabia 2006), required time and temperature are lower. On the other hand, it should be search for microorganisms, which are able to decompose biodegradable plastics at temperatures as low as possible. Due to growing pollution of the world, it is necessary to improve the biodegradation process of bioplastic by reduction of the time or by decreasing the temperature required for an efficient process. Moreover, by finding microorganisms which can assimilate many types of bioplastics it is possible to make the biodegradation process more efficient.

The aim of this study was to investigate the natural properties of microorganisms isolated from extreme environments for degradation of bioplastic.

## Materials and methods

### Materials

Fifty-two soil samples were collected along western and central parts of Spitsbergen, Svalbard Archipelago (77.5266°N, 14.7577°E; 77.5302°N, 13.9291°E; 77.5141°N, 14.5638°E; 77.54°N, 14.5675°E) at the turn of July and August 2014. All samples were kept in sterile falcons, transported with precautions and stored in cooling conditions (4 °C). PBSA (Bionolle 3020MD) and PBS (Bionolle 1020MD) pellets were purchased from Showa Denko K.K. (Japan). PLA pellets were obtained from BIOMAR (Germany). PCL pellets were purchased from TRESNO (Poland). PLA films (Earthfirst PLA) were supplied by Pakmar Sp. z o.o. (Poland). PCL bags were supplied by BioBag (Poland). Commercially available shopping bags made from corn and potato starch were purchased at Carrefour. Sarkosil NL, used to prepare emulsions, was purchased from Sigma-Aldrich (Germany).

### Preparation of polymer emulsion and biopolymer films

0.5% PBSA emulsion was prepared as follows: a 2 g PBSA pellet was dissolved in 40–60 ml of dichloromethane. 100 ml distilled water and 2 ml of 2% Sarkosil NL were added. The mixture was sonicated (10 min). After sonication (Sonics Vibra Cell VCX500), dichloromethane was evaporated by stirring at 80 °C for 2 h in a draft chamber. The emulsion was filled up to 400 ml with distilled water. The pH was adjusted to 7 with KOH. All the other polymer emulsions (PBS, PCL and PLA) were prepared using the same procedure (Uchida et al. 2000).

The polymer films were cut into small squares (2 cm × 2 cm) and were sterilized using 70% ethanol and UV radiation (5 min) (Li et al. 2012).

### Media

A mineral minimum medium [MM] containing 0.1% emulsified polymers was prepared for isolation and examination of BP-degrading microorganisms. The composition was as follows: 0.2% NaH_2_PO_4_, 0.05% MgSO_4_·7H_2_O, 0.02% KH_2_PO_4_, 0.1% yeast extract and 0.1% polymer emulsion (PBSA, PBS, PCL or PLA) as the only source of carbon for the growth of bacteria (Kitamoto et al. 2011). To solidify the medium, agar (2%) was added. LB agar medium, composed of 1% tryptone, 0.5% yeast extract, 0.5% NaCl, and 2% agar, was used for bacteria isolation. Martin agar, contained 0.5% peptone, 0.1% K_2_HPO_4_, 0.05% MgSO_4_·7H_2_O, 1% glucose, 30 mg/l Bengal rose and 2% agar was used for fungi isolation.

### Isolation of microorganisms

1 g of soil sample was transferred to a flask containing 50 ml of sterile 0.85% physiological saline. The soil solution was shaken for 1 h on a rotary shaker (Sartorius Certomat MOII) at 37 °C. The culture (100 µl) was then spread on Martin and LB agar plates to isolate microorganisms. The plates were incubated at 28 °C for 5–7 days in a bacteriological incubator (Memmert). The developed colonies were isolated and sub-cultured repeatedly to obtain the pure cultures and then preserved in agar plates at 4 °C.

### Clearance method

Plastic-degrading microorganisms were identified by zone of clearance method. The microbial colonies cultivated on plates were tested on mineral minimum [MM] agar plates containing 0.1% emulsified polymers. The bacterial biomass was inoculated as a scratch on agar plates. After incubation (2–3 days, 28 °C), the clear zones were observed. The colonies forming clear zones were selected as BP-degrading strains for further analysis.

### Standardization tests

The degradation of BP was further analyzed by measuring the ability to clear zones formation in wells. In these assays 5 wells were made in minimal medium agar plates. Next, 100 µl of liquid culture (OD_600_ = 1.0) of the isolated microorganisms was applied in the wells. The control was 100 µl of sterile water. During incubation (28 °C) after 3, 5 and 7 days, the clear zones were observed and diameters were measured. It was allowed to accurate comparison the activity between isolated microorganisms. Strains with the largest zones were selected for identification.

### Plate tests for fungi

The fungal biomass was tested on MM agar plates without additional emulsions. The liquid cultures of fungi were plated onto the minimum medium, which was subsequently covered with bioplastic films. Bioplastic films of PLA, HDPE, corn and potato starch, and PCL were used. The control was plate without inoculation of the cell suspension. All plates were incubated at 28 °C for 3 days, then plates were removed to room temperature (20 °C) and stored for 21 days. Growth of fungi strains on surface of films were determined and averaged, and their degradation rates were evaluated according to the following 5 class index: 0–0% degradation; 1—less than 10% degradation; 2—10 to 30% degradation; 3—30 to 60% degradation; and 4—over 60% degradation (Bajer and Kaczmarek 2007). Strains and plastic films with the highest degradation rates were chosen for the liquid culture method.

### Liquid culture method for fungi

Approximately 0.1 g of plastic films (squares 1 cm × 1 cm) was aseptically transferred into the conical flask containing 100 ml of sterile mineral minimum medium. Films were sterilized by washing in 70% ethanol and exposing in UV radiation for 5 min. Then medium was inoculated with the selected strain incubated in rotary shaker at 28 and 21 °C, 220 rpm (Sartorius Certomat MOII) for a 1-month period. The control was maintained with films in a microbe-free medium (Usha et al. 2011).

### Analysis of degradation of plastic films

At the end of the process bioplastic films were collected, washed thoroughly using distilled water and shade dried (Usha et al. 2011). Degradation of PCL and starch films was determined by measuring the residual weight of the polymers. The percentage weight change was calculated by comparing the dry weight of residual films with the original weight of the films (Prema et al. 2013) using the formula (Kyaw et al. 2012):

$${\text{Weight loss}} \left( \% \right) = \frac{{{\text{Initial weight}} - {\text{Final weight}}}}{\text{Initial weight}} \times 100$$

Scanning electron microscopy was performed to characterize surface morphology. At the end of the flasks-shake cultivation, the films were recovered washed in sterile water and dried. Next, the films were coated with gold (Kressington 108 a sputter coater) and were observed under a VEGA TESCAN 3 scanning electron microscope (Tescan, USA) at an acceleration voltage of 5 kV.

### Identification of isolated strains

The microorganisms isolated from soil were identified by 18S rRNA and 16S rRNA sequence analysis (Gajendiran et al. 2016). gDNA was isolated from pure cultures of fungi and bacteria. The fungal DNA sequences were obtained by Gene Matrix Plant & Fungi DNA Purification Kit (EURx, Poland). In this kit, the temperature shock method was used to break open the cellulosic cell wall to obtain DNA. The 18S rRNA sequence was amplified by polymerase chain reaction. The primers used for identification of fungi were nu-SSU-0817-59 (5′-TTAGCATGGAATAATRRAATAGGA-3′), nu-SSU-1196-39 (5′-TCTGGACCTGGTG AGTTTCC-3′), and nu-SSU-1536-39 (5′-ATTGCAATGCYCTATCCCCA-3′). The bacterial genomic DNA was isolated by Genomic Mini Kit for isolation of DNA from bacteria, cell cultures and tissues (A&A Biotechnology, Poland). The bacterial 16S region, after amplification, was identified by using 5′-GATTAGATACCCTGGTAG-3′ and 5′-AGTCACTTAACCATACAACCC-3′ as primers. The primers for fungal and bacterial sequencing have been described previously (Borneman and Hartin 2000; Magray et al. 2011). The sequencing result was compared by BLAST search tool in the GeneBank National Centre for Biotechnology Information (NCBI) database. The nucleotide sequences have been added into the database (GenBank) and the accession numbers are provided in Table 3. The strain *Clonostachys rosea* 16G was deposited in Collection of Industrial Microorganisms (IAFB), Warsaw, Poland under number KKP 2300.

## Results

### Isolation of microorganisms

In extreme environments microorganisms have confirmed many unique features which help them to survive under unfavorable conditions. We focused on the Arctic environment to isolate microorganisms which are able to rapidly decompose bioplastics at low temperatures. In total, 52 soil samples were collected from glacial, periglacial and coastal environments of Spitsbergen. The soil samples were transferred to flasks containing sterile saline and then were shaken (for details see “Materials and methods”).

During the isolation of microorganisms, 313 microorganisms were chosen for further studies: 289 bacterial and 24 fungal strains. The pure cultures of microorganisms were stored at cooling conditions until the start of the plate tests.

**Fig. 1:**
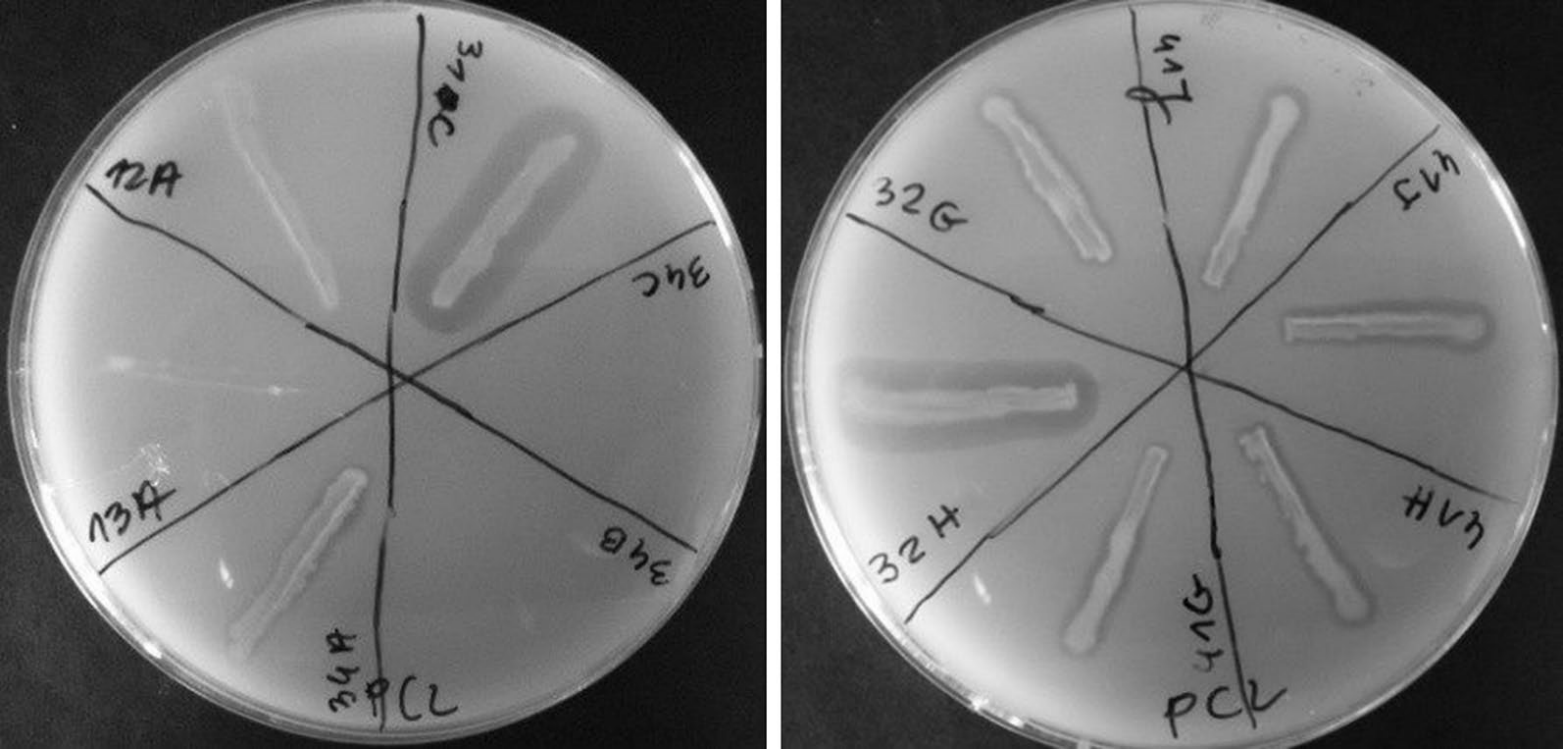
Example of an initial test of biodegradability

The main aim of this study was investigation of the decomposing abilities of polar microorganisms. Therefore, the isolated microorganisms were screened for PLA, PCL, PBS, and PBSA degradation by the clear zone method. The clear zone was shown by 113 bacteria (39.10%) and by 8 fungi (33.33%) after 2, 3 and 7 days. The microorganisms forming clear zones were selected as BP-degrading (PLA, PBS, PBSA or PCL) (Fig. 1). The results for each bioplastic are shown in Table 1.

**Table 1:** Microorganisms degrading plastics: PBS, PBSA, PCL and PLA

Microorganism	PBSA		PBS		PCL		PLA^a^	
	Number	%	Number	%	Number	%	Number	%
Bacteria	106	93.81	65	57.52	94	83.19	51	45.13
Fungi	10	90.91	8	72.73	8	72.73	6	54.55

^a^Measured growth of microorganisms. Clear zones were not observed

### Standardization tests for bacteria

**Fig. 2:**
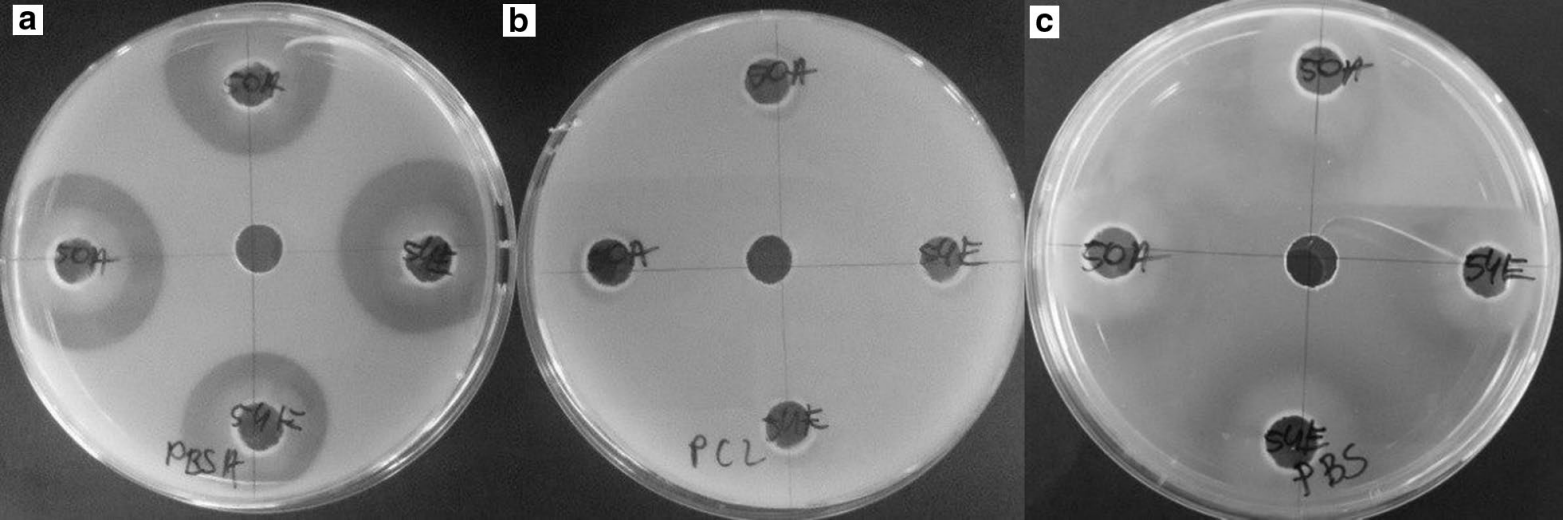
Comparison of clear zones of the same microorganisms on medium containing 0.1% PBSA (**a**), 0.1% PCL (**b**), 0.1% PBS (**c**)

The next step in our study was selection of microorganisms which possess the best biodegradation properties. Thus, microorganisms that formed a clear zone on emulsified plates were chosen for the standardization test. The clear zones around the wells were measured after 24, 48, 72, 144 and 172 of incubation (Fig. 2). At the end of the experiment, bacterial strains which showed the widest clear zone on at least 2 types of emulsion were chosen for identification. The minimal size of the clear zone chosen for the experiment was PBSA 16 mm, PBS 12 mm, and PCL 15 mm, and growth on PLA plates was 10 mm. Based on this result, 12 bacterial strains were selected and named as 4A, 52G, 31C, 42C, 42D, 42E, 5D, 31A, 28E, 2B, 33C, and 23B.

### Plate tests for fungi

**Fig. 3:**
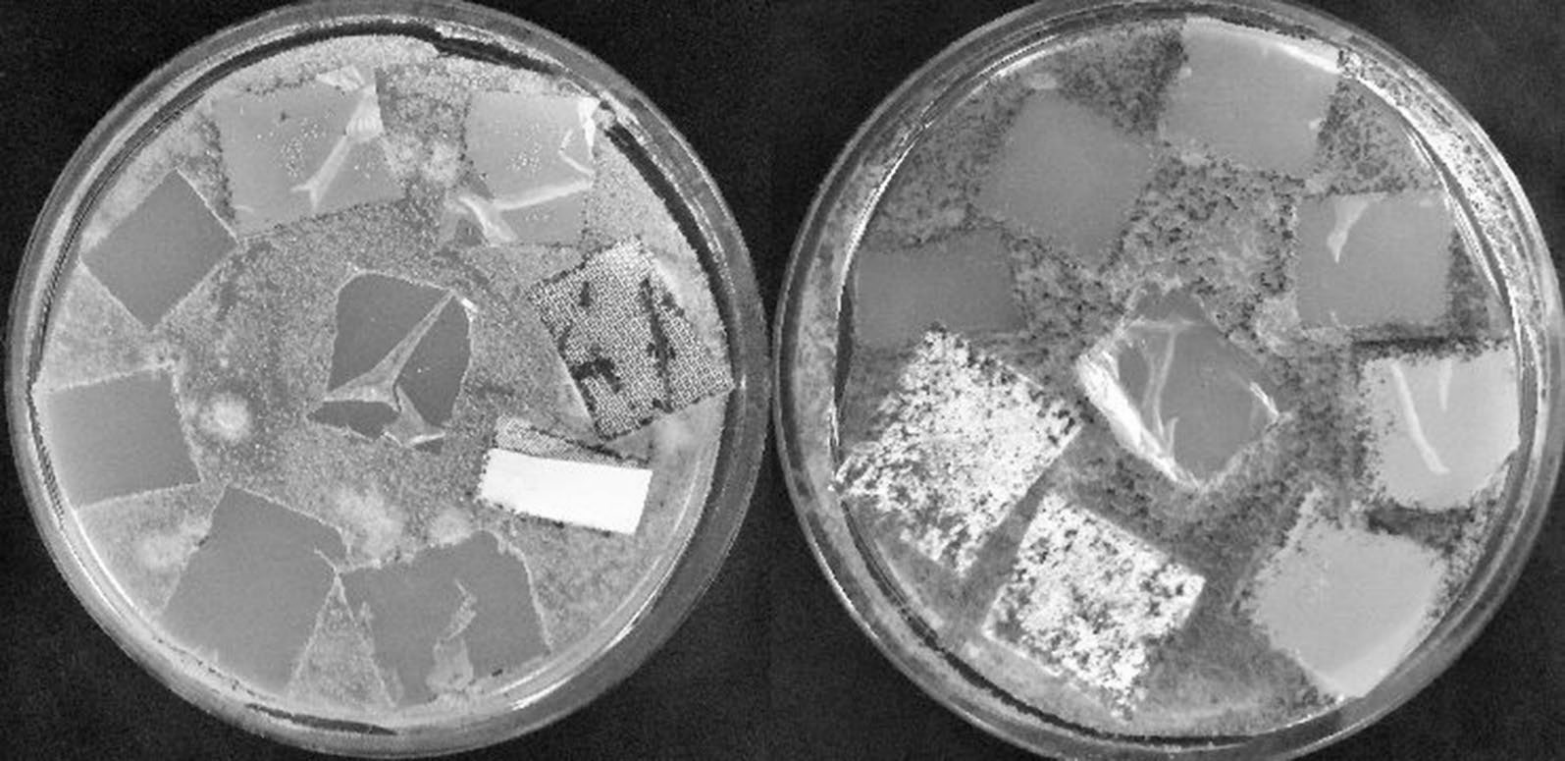
Agar plate test for fungi

In the clearance method test, eight fungal strains showed high abilities in emulsion degradation. Therefore, to verify which type of bioplastic is the most suitable for the assays, additional Petri dish tests with plastic films were conducted for eight fungal strains (named 16G, 43C, 16H, 54C, 50A, 21K, 28K, and 28J). All strains were plated out onto appropriate medium (for details see “Materials and methods”) and incubated. After 21 days of incubation, the highest ability to grow on films was noted for fungi 16H and 16G. These two strains showed the highest degree of biodegradation with starch films and PCL films (Fig. 3); therefore they were chosen for the further studies. In the 5-class index the PCL films were degraded by 16G at level 3 and 16H at level 4. Starch films were degraded by both strains at level 2. These were the most promising results achieved on the agar plates among the various types of films. Given these results, these two types of bioplastic (starch and PCL films) were chosen for the liquid culture method.

### Determination of degradation bioplastic films by fungi

**Fig. 4:**
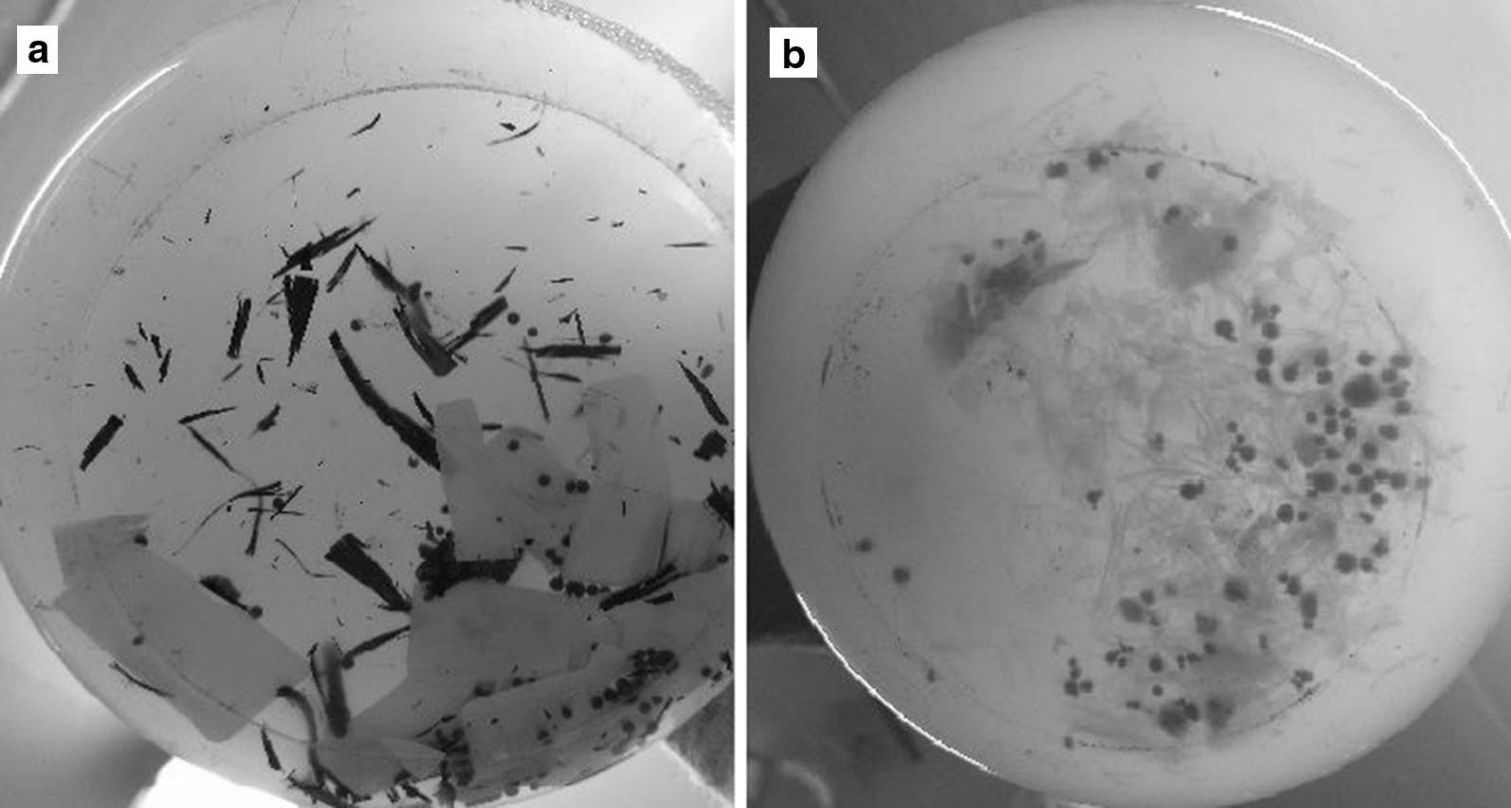
PCL (**a**) and starch (**b**) films at the end of cultivation at 20 °C by 16G

**Fig. 5:**
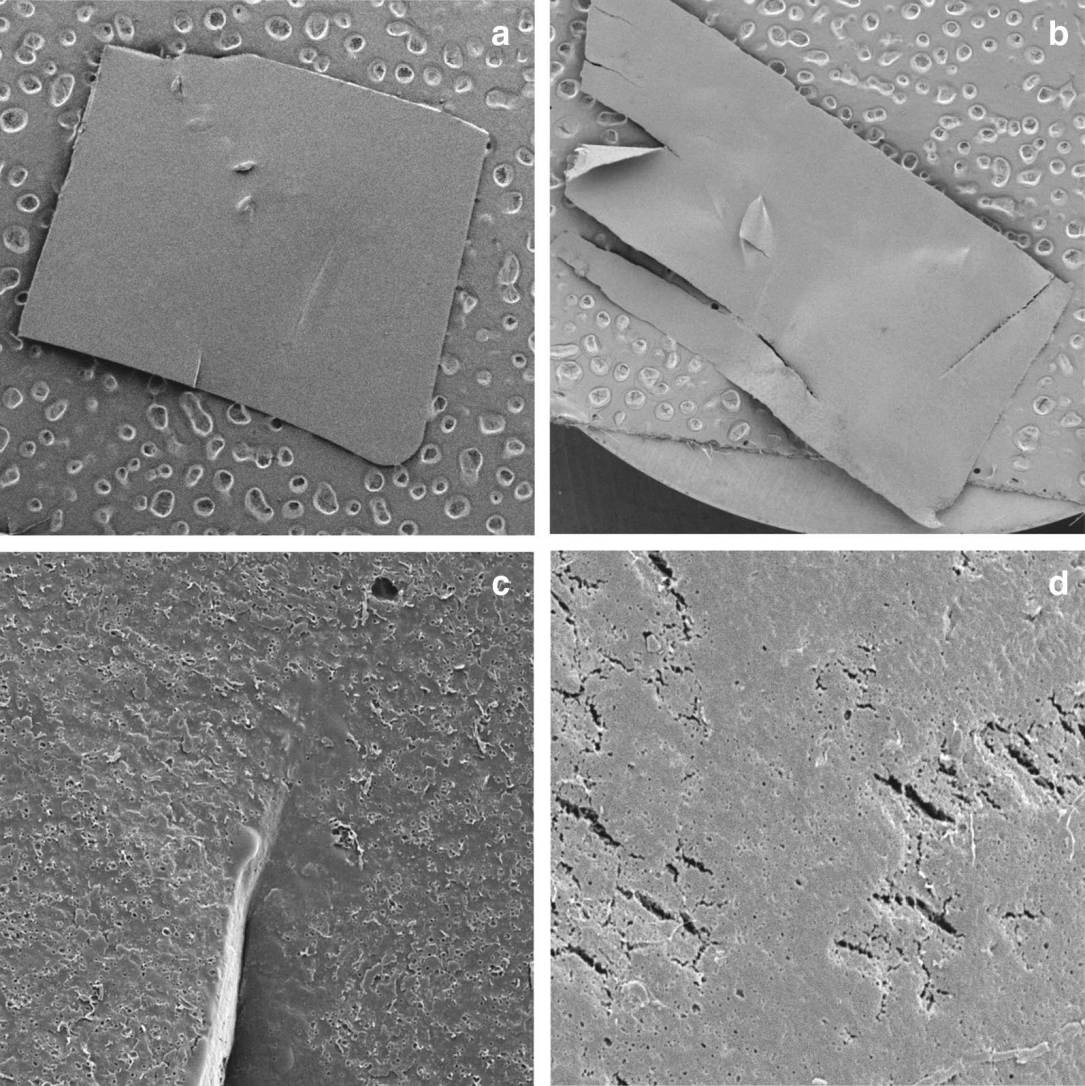
PCL films at the end of cultivation at 20 °C by *C. rosea* 16G. The films were viewed by scanning electron microscopy at a 40 magnification (**a**, **b**) and at a 2000 magnification (**c**, **d**), as described in “Materials and Methods”. **a**, **c** The control; **b**, **d** *C. rosea*

**Fig. 6:**
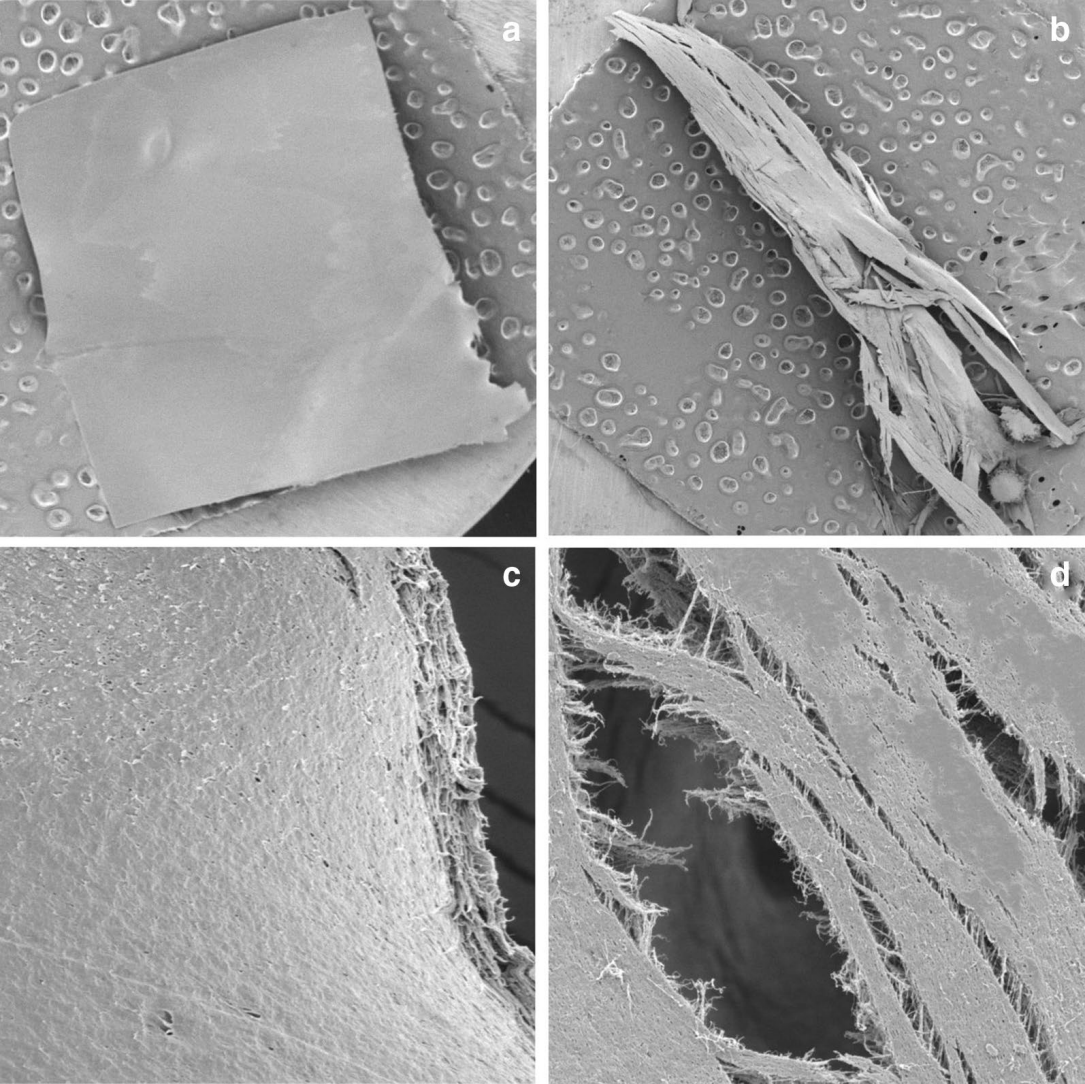
Starch films at the end of cultivation at 20 °C by *C. rosea* 16G. The films were viewed by scanning electron microscopy at a 40 magnification (**a**, **b**) and at a 2000 magnification (**c**, **d**), as described in “Materials and Methods”. **a**, **c** The control; **b**, **d** *C. rosea*

To confirm the abilities of the selected strains for degradation of biodegradable films, the liquid culture method was performed. The experiment took 30 days, and the results are summarized in Table 2. During this period, the degree of film degradation was carefully observed. Interestingly, strain 16G was able to decompose the film sooner. It started decomposing starch films after 8 days of incubation, and within 16 days the decomposition reached 100%. At the end of the experiment PCL films were 52.91% decomposed (w/w). Due to high efficiency of 16G at 28 °C, the culture method was repeated at a lower temperature (21 °C). Strikingly, strain 16G was able to decompose starch film by 65% (w/w) and PCL film by 34.5% within 30 days (Fig. 4). During cultivation at 21 °C faster decomposition of PCL films than starch films was observed. An opposite phenomenon was observed at 28 °C. Strain 16H showed lower ability to degrade bioplastics at 28 °C. It decomposed PCL films by 21.54% and starch films by 12.07%. At the end of the experiment, the PCL and starch films were tested by SEM for the examination of the surface erosion. As seen on Figs. 5 and 6, strain 16G showed high degrading activity. Physical changes, such as the formation of pits and cracks on the surface were observed. It is worth noting that the important factors stimulating biodegradation were limitation of the carbon and nitrogen source and the aeration rate. When cultures were grown in bottles (limited supply of oxygen) with the addition of a nitrogen source, degradation was inhibited (data not shown). However, culturing strains in flasks with complete absence of additional carbon and nitrogen source stimulated the biodegradation process by the isolated fungi.

**Table 2:** Summary of biodegradability of filamentous fungi after 30 days liquid culture method in 28 °C

Starch film		PCL film	
Strain	%	Strain	%
16H	12.07	16H	21.54
16G	100^a^	16G	52.91
16G^b^	65.00	16G^b^	34.50

The weight loss of film fragments expressed in % (w/w)

^a^Decomposed after 16 days of growth

^b^Liquid culture conducted at 20 °C

### Identification of isolated microorganisms

Next, we sought to identify the isolated strains of bacteria and fungi. Thus a comparison of the 16S RNA and 18S RNA sequence with known sequences at NCBI was done. Isolated bacteria were identified as belonging to *Pseudomonas* species (2B, 5D, 28E, 42D, 42E, 42C, 52G as *Pseudomonas* sp., 4A, 31A as *Pseudomonas frederiksbergensis*, 33C as *Pseudomonas mandelli*) and *Rhodococcus* species (23B). Fungal strains were identified as *C. rosea* (strain 16G) and *Trichoderma* sp. (strain 16H) (Table 3). The 16S/18S rRNA gene sequences of the isolated bacterial and fungi were submitted to Genbank under the accession numbers presented in Table 3.

**Table 3:** Microorganisms able to biodegradation process isolated from Spitsbergen soil samples

No.	Strain	Similarity (%)	Species	Accession number
1.	2B	96	*Pseudomonas* sp.	MF034061
2.	4A	98	*Pseudomonas frederiksbergensis*	MF034062
3.	5D	96	*Pseudomonas* sp.	MF034063
G4.	23B	97	*Rhodococcus* sp.	MF034064
5.	28E	97	*Pseudomonas* sp.	MF034065
6.	31A	98	*Pseudomonas frederiksbergensis*	MF034066
7.	31C	96	*Pseudomonas* sp.	MF034067
8.	33C	99	*Pseudomonas mandelii*	MF034068
9.	42C	94	*Pseudomonas* sp.	MF034069
10.	42D	96	*Pseudomonas* sp.	MF034070
11.	42E	96	*Pseudomonas* sp.	MF034071
12.	52G	96	*Pseudomonas* sp.	MF034072
13.	16G	99	*Clonostachys rosea*	MF034074
14.	16H	99	*Trichoderma* sp.	MF034074

## Discussion

Currently, the growing quantity of plastic wastes has become one of the most important pollution/environmental issues in the world. The solution of this problem may be an improvement of the biodegradation process. Microbial activity can be used instead of the conventional methods. Researchers are searching for microorganisms that can effectively decompose huge quantities of plastic wastes. Research is focused on unconventional and wide-range resources; for this reason microorganisms are isolated from littered areas, but also from unusual places, such as the depths of the seas (Sekiguchi et al. 2010), gastric juices of worms (Yang et al. 2014) and the surface of leaves (Kitamoto et al. 2011). Searching for microorganisms in various, untypical environments is surprisingly effective, therefore the research should be conducted for both conventional plastic and bioplastic.

Microorganisms isolated from specific environments have developed many unique features which help them to survive under unfavorable conditions. For this reason, we focused on the Arctic environment to isolate microorganisms which are able to rapidly decompose bioplastics at low temperatures, what can reduce electric energy usage in biodegradation process in laboratory conditions.

In the present study, we sought to isolate, test and identify Arctic microorganisms which possess high ability for biodegradability. After pilot scratch assays with 313 microorganisms, we isolated 113 bacteria and 8 filamentous fungi (in total 121 microorganisms) degrading PBSA, PBS, PCL or PLA on agar plates. In total 116 (95.87%) microorganisms degraded PBSA emulsion, 73 (60.33%) PBS, 102 (84.3%) PCL and 56 (46.28%) grew on PLA emulsion. In other studies (Penkhrue et al. 2015) there were isolated 26 (32.9%) strains degrading PLA, 44 PBS (55.7%) and 58 PCL (73.4%) from soil samples collected in northern Thailand. Degradation of PBSA emulsion was not tested; in our study it reached the highest degree of biodegradation. Despite this fact, medium with PCL as a carbon source was degraded the easiest, while in contrast PLA was the hardest to degrade. In the case of PLA, the growth of microorganisms was evaluated without zones of clearance. Teeraphatpornchai et al. (2003) noted that PLA-degrading microorganisms always show biodegradable activity with other bioplastics, but most PBSA- or PBS-degrading microorganisms do not have the ability to degrade PLA. In that research, among 400 soil samples, only 4 strains showed clear zones with PLA emulsion, but only 1 strain (identified as *Paenibacillus amylolyticus*) was effectively degrading PBSA, PBS and PCL as well. Next, in the samples from the bottom of the Pacific Ocean 13 bacterial strain were identified, as *Shewanella* sp., *Moritella* sp., *Psychrobacter* sp. and *Pseudomonas* sp. (Sekiguchi et al. 2010). Interestingly, these bacteria showed clear zones only on PCL emulsion but not on other bioplastics such as PHB, PBS, PBSA or PLA. The lack of clear zones on PLA plates could be caused by too dry environmental conditions. It was shown that biodegradation of PLA occurs in a water environment or damp setting, which is difficult to replicate long-term in plate tests (Nowak and Pająk 2010). Additionally, PLA is a type of polymer which needs more time to be degraded. It was noted, that in compost where the temperature was in the range 50–60 °C and with high humidity, degradation of PLA lasts 60 days. Among 5 PLA-degrading strains isolated from compost, after 25 days at 37 °C culture, the highest degradation (45.5%) was shown by strain MS-2 identified as *Bacillus amyloliliquefaciens* (Prema et al. 2013). In our experiment lower temperature was used, therefore it might explain lack of the clearance zones on plates with emulsified PLA.

In our study, the bacteria with the highest degradation ability were identified as *Pseudomonas* sp. and *Rhodococcus* sp. The abilities of *Pseudomonas* sp. for biodegradation were described previously (Sekiguchi et al. 2010; Usha et al. 2011), *Pseudomonas putida* (Saminathan et al. 2014), *Pseudomonas aeruginosa* (Asmita et al. 2015; Nirmala and Harini 2014). According to our knowledge, *Rhodococcus* sp. isolated in this study had not been reported before as a BP (biodegradable plastics)-degrading microorganism. *Rhodococcus* sp. showed high degradation activity against emulsified PCL, similar to isolated *Pseudomonas* sp. The emulsified PCL seems to be the easiest type of bioplastic for bacteria to degrade.

For fungal strains we conducted plate tests with four different types of BP-plastic films—PLA, PCL, starch and HDPE—as the sole carbon source. After 21 days of growth, we choose starch and PCL films as the most accessible type of BP plastics for fungal biodegradable activity. These type of plastics were tested in the liquid culture method with the most efficient strains, identified as *Trichoderma* sp. and *C. rosea* selected in this experiment.

In the liquid culture method *Trichoderma* sp. degraded starch films by 12.07% and PCL by 21.54% per month at 28 °C. The biodegradation activity of *Trichoderma* sp. was described previously; it was reported that a high level of growth on PCL film was characterized by the species *Trichoderma viride* (Janczak et al. 2014). *Clonostachys rosea* degraded 100% of starch films during 16 days and 52.91% of PCL film during 30 days at 28 °C. At 21 °C this fungus degraded 65% of starch and 34.50% of PCL films within 30 days. To date, this is the first report on starch and PCL degradation by *C. rosea* showing effective ability of degradation by this species. Moreover, our research proves that PCL is a well biodegradable plastic. Previously, similar results were obtained: *Penicillium oxalicum* DSYD05-1 degraded PCL films during 10 days, but incubation was conducted at 30 °C (Li et al. 2012). In another study biodegradation of PCL films and PCL foam plastic by *Pseudozyma japonica* Y7-09 was evaluated. PCL films were degraded by 93.33% after 15 days of cultivation at 30 °C and PCL foam by 43.2% per month (Fatma et al. 2014).

Hayase et al. tested the biodegradation ability of the bacterium *Bacillus pumilis* 1-A isolated from soil. PBSA, PBS, PBS/PCL blend and PLA were tested in 14-day cultivation at 30 °C. The loss of bioplastic films weight was obtained as: 100% PBSA, 90.2% PBS, 50.8% PBS/PCL and 0.9% PLA (Hayase et al. 2004). In the study with process optimization *Aspergillus versicolor* was able to degrade PBSA films by 90% after 25 days of cultivation at 30 °C (Zhao et al. 2005). These data suggest that *C. rosea* might decompose these films efficiently after process optimization.

In summary the Arctic environment is a good source of microorganisms able to undergo the biodegradation process. Their main advantage is the ability to grow at low temperature and efficient biodegradation under this condition. Bioplastics might be a good carbon source for some microorganisms. In this study we found that bioplastics are degraded in a short period of time by *C. rosea*. These data suggest that isolated microorganisms can be used in natural and laboratory conditions for degradations of BP. Moreover, further optimization of growth conditions such as co-culture of both fungi and bacteria might significantly accelerate the process of decomposition of plastics. To clarify the degradation mechanism in more detail, research on the produced enzymes should be conducted.

## Abbreviations

BP: biodegradable plastics
PCL: poly(ɛ-caprolactone)
PLA: poly(lactic acid)
PBS: poly(butylene succinate)
PBSA: poly(butylene succinate-co-butylene adipate)
